# Deep Learning‐Based Classification of Histone–DNA Interactions Using Drying Droplet Patterns

**DOI:** 10.1002/smsc.202400252

**Published:** 2024-08-10

**Authors:** Safoura Vaez, Bahar Dadfar, Meike Koenig, Matthias Franzreb, Joerg Lahann

**Affiliations:** ^1^ Institute of Functional Interfaces (IFG) Karlsruhe Institute of Technology (KIT) Hermann‐von‐Helmholtz‐Platz 1 76344 Eggenstein‐Leopoldshafen Germany; ^2^ Biointerfaces Institute Departments of Chemical Engineering, Materials Science and Engineering, and Biomedical Engineering, and the Macromolecular Science and Engineering Program University of Michigan Ann Arbor MI 48109 USA

**Keywords:** coffee ring, deep learning image analysis, drying droplet, histone–DNA interaction

## Abstract

Developing scalable and accurate predictive analytical methods for the classification of protein‐DNA binding is critical for advancing our understanding of molecular biology, disease mechanisms, and a wide spectrum of biotechnological and medical applications. It is discovered that histone–DNA interactions can be stratified based on stain patterns created by the deposition of various nucleoprotein solutions onto a substrate. In this study, a deep‐learning neural network is applied to categorize polarized light microscopy images of drying droplet deposits originating from different histone–DNA mixtures. These DNA stain patterns featured high reproducibility across different species and thus enabled comprehensive DNA categorization (100% accuracy) and accurate prediction of their respective binding affinities to histones. Eukaryotic DNA, which has a higher binding affinity to mammalian histones than prokaryotic DNA, is associated with a higher overall prediction accuracy. For a given species, the average prediction accuracy increased with DNA size. To demonstrate generalizability, a pre‐trained CNN is challenged with unknown images that originated from DNA samples of species not included in the training set. The CNN classified these unknown histone‐DNA samples as either strong or medium binders with 84.4% and 96.25% accuracy, respectively.

## Graphical abstract

Stain patterns of nucleoprotein solutions exhibit high reproducibility across species: Controlled drying of the solutions droplet on a surface reveals histone–DNA relative binding affinity. A pre‐trained network on polarized light images of various stains accurately predicts both DNA samples and the binding trends of unknown histone–DNA samples that were not included in the training image set.

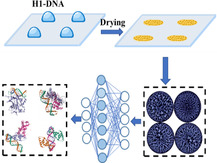

## 1 Introduction

Interactions between DNA and proteins are implicated in almost all aspects of life.^[^ ^1^, ^2^, ^3^ ^]^ Methods to analyze protein–DNA interactions have a wide range of applications in various fields of science and technology including drug discovery and development,^[^ ^4^ ^]^ diagnostics medicine,^[^ ^5^ ^]^ biotechnology and genetic engineering,^[^ ^6^ ^]^ genomic research and regulation,^[^ ^7^, ^8^ ^]^ and neuroscience.^[^ ^9^ ^]^ Contemporary methods include vivo techniques such as chromatin immunoprecipitation (ChIP),^[^ ^10^ ^]^ electrophoretic mobility shift assay (EMSA),^[^ ^11^ ^]^ SELEX‐based techniques^[^ ^12^ ^]^ southwestern blotting,^[^ ^13^ ^]^ and biophysical techniques like fluorescence‐based techniques,^[^ ^13^ ^]^ circular dichroism,^[^ ^13^ ^]^ atomic force microscopy,^[^ ^13^ ^]^ nuclear magnetic resonance (NMR),^[^ ^14^ ^]^ surface plasmon resonance spectroscopy,^[^ ^13^ ^]^ and X‐ray crystallography.^[^ ^13^, ^15^ ^]^ However, these methods have several limitations including the need for 1) specific and potentially expensive antibodies that are not widely accessible (e.g., ChIP), 2) multimodal analytics (e.g., NMR), 3) elaborate and time‐consuming sample preparation (e.g., SELEX‐based methods), or 4) the need for specialized equipment and facilities (e.g., X‐ray techniques).^[^ ^13^, ^16^, ^17^ ^]^ Therefore, it is still a challenge to find and develop fast, simple, inexpensive, and accurate methods to characterize protein–DNA binding affinities.^[^ ^18^ ^]^ In previous studies, the preferential interaction of linker histone (H1) with eukaryotic DNA was contrasted to prokaryotic DNA through binding assays utilizing nitrocellulose filters.^[^ ^19^ ^]^ Al‐Natour et al. (2007) showed the binding of highly lysine‐rich H1 to superhelical DNA, favoring it over linear or nicked circular DNA forms as deduced from direct competition experiments.^[^ ^20^ ^]^ Lymphocyte DNA fragments, weighing 2 × 10^6^ Da exhibited a binding affinity with H1 at a magnitude at least 15 times greater than equivalent E. coli fragments of the same molecular weight.^[^ ^21^ ^]^ Other studies demonstrated that helix and turn formation can be induced in a peptide from the COOH‐terminal domain of histone H1 by binding to double‐stranded DNA.^[^ ^22^ ^]^ Luiza et al. found that the vibrational characteristics of DNA, notably PO_2_^−^, were influenced in distinct ways by binding to histone H1, protamine, and histone‐mimicking macromolecules.^[^ ^23^ ^]^ Specifically, they reported that the shift of DNA's PO_2_^−^ antisymmetric stretching bands were characteristically altered by the presence of lysine‐rich histones.^[^ ^23^ ^]^ Alternatively, molecular dynamic (MD) modeling techniques can predict the binding affinity of biomacromolecules.^[^ ^24^ ^]^ Despite the multitude of studies focusing on protein–ligand and protein–protein docking simulations,^[^ ^25^ ^]^ MD methods still have limitations for DNA or larger proteins.^[^ ^25^, ^26^, ^27^ ^]^ Machine learning (ML) approaches have been discussed as alternatives,^[^ ^28^ ^]^ but require access to large data sets.^[^ ^29^ ^]^ While the protein data bank (PDB) contains over 219 869 experimentally determined structures as of May 2024, the number of protein–DNA complex structures listed is limited to several thousands.^[^ ^30^ ^]^

Over the last few decades, increasing attention has been directed toward the patterns obtained from drying droplets.^[^ ^31^, ^32^, ^33^ ^]^ Experimentally, characteristic patterns can emerge from drying droplets of biological fluids containing nonvolatile species, which reflects the physicochemical dynamics in droplet wetting and evaporation.^[^ ^34^ ^]^ The phenomenon sometimes referred to as the “coffee‐ring effect” depends on various parameters, such as environmental conditions (temperature, relative humidity), nature of salute components (chemical composition, size, and initial concentration),^[^ ^35^ ^]^ and the substrate chemistry.^[^ ^36^, ^37^ ^]^ Askounis et al. (2016) indicated that lower viscosity in droplets containing shorter DNA chains facilitates easier mobility and continuous deposit growth, causing dendrite crystal formation through diffusion‐limited growth. Conversely, for longer DNA chains, crystallization was attributed to “faceted growth”, primarily a nucleation‐limited process.^[^ ^38^ ^]^ Some other examples of published deposit patterns include uniform films,^[^ ^39^ ^]^ fingering‐like pattern,^[^ ^40^ ^]^ dendritic pattern,^[^ ^41^ ^]^ dot‐like pattern,^[^ ^42^ ^]^ as well as more complex arrangements.^[^ ^37^, ^43^ ^]^

In our previous study, we delved into the development of ML‐based image analysis methods for predicting single amino acid mismatches in proteins using deep learning techniques. We showed that crucial insights about both primary and secondary peptide structures could be gathered from the patterns of residues left by drying droplets. We used deep‐learning neural networks trained with polarized light microscopy images obtained from the drying droplet deposits of various amyloid‐beta peptides to analyze complex stain patterns.^[^ ^44^ ^]^

Here, we leverage conventional image‐based neuronal networks for the analysis of large cohorts of images obtained by drying histone–DNA solutions. We developed an automated workflow that involves depositing defined volumes of histone–DNA complexes in a massively parallel fashion and obtaining images of the stain patterns using an automated polarized light microscope. This workflow resulted in thousands of images within a couple of hours. This cost‐efficient, fast, and automated method to study histone–DNA interactions has been successfully applied to both eukaryotic and prokaryotic DNA samples.

## 2 Results and Discussion

**Figure 1:**
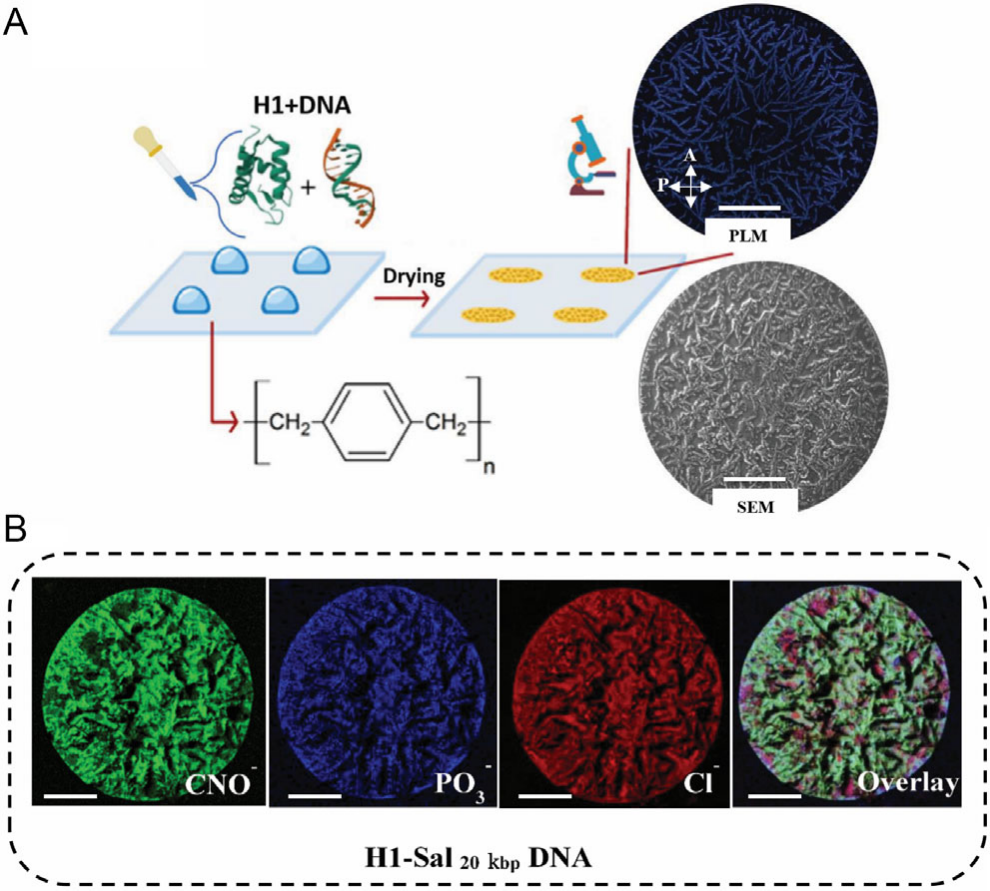
Deposition stains of H1‐DNA droplets reveal complex information about protein‐DNA interactions. Stains were obtained by depositing 2 μL droplets of an aqueous HEPES buffer solution onto hydrophobic poly (para‐xylylene) coated glass wafers. A) Representative PLM and SEM images of the same dried stain obtained from an H1–Sal_20 kbp_ DNA mixture revealing complex deposition patterns. The scale bars represent 500 μm. B) Analysis of a H1–Sal_20 kbp_ DNA mixture stain via TOF‐SIMS imaging reveals the presence of PO_3_^−^ (intensity color scale 0–2 counts), highlighted in blue. The amino acids of histone and the nucleotides of DNA, are distinguished by CNO^−^ fragments (marked in green, intensity color scale 0–30 counts). The chloride ions distribution stemming from the buffer solution is displayed in red (intensity color scale 0–30 counts). First row: RGB channels and their overlay. The scale bars represent 500 μm.

To create uniform and hydrophobic substrates, which are crucial for ensuring consistent circular droplets with an average water contact angle (80 ± 1°) across large surface areas, we utilized chemical vapor deposition (CVD) polymerization. Specifically, we used CVD polymerization to coat glass wafers with homogenous poly(*p*‐xylylene) (PPX) films of 50 nm thickness.^[^ ^45^ ^]^ Subsequently, a defined and constant volume of 2 μL of an aqueous solution containing the histone–DNA mixture was dispensed onto the coated surfaces and allowed to dry under controlled humidity and temperature conditions (40%, 23 °C) for 50 min. Throughout the study, the same buffer was used which contained 100 mM 4‐(2‐hydroxyethyl)‐1‐piperazineethanesulfonic acid (HEPES) buffer (pH 7.8), 150 mM potassium chloride, and 50 mM ammonium sulfate.^[^ ^46^ ^]^ Potassium chloride and ammonium sulfate were included because of their kosmotropic properties, i.e., they prevent protein denaturation and enhance salting‐out effects promoting protein–DNA interactions.^[^ ^47^, ^48^ ^]^ The patterns created during deposition from protein–DNA mixtures are compositionally straightforward, but structurally intricate supramolecular systems that are regulated by locally and temporally interconnected multiscale processes.^[^ ^33^ ^]^ As the protein–DNA solution dries, the solution becomes saturated until its components ultimately precipitate. Salt and biomolecule deposition initiates at the periphery of the droplet and progresses toward central droplet regions leading to at least partially crystalline stains (Video S1, Supporting Information). We thus routinely characterized these deposition patterns with polarized light microscopy (PLM). **Figure** **1** displays a PLM image depicting a typical drying pattern obtained from a histone‐Salmon_20 kbp_ DNA mixture. For topographic characterization of the deposited patterns, we employed scanning electron microscopy (SEM). The SEM image revealed the existence of high‐aspect structures, which are distributed throughout the stain pattern (Figure 1A).

For chemical characterization, we utilized time‐of‐flight secondary‐ion mass spectrometry (TOF–SIMS) as illustrated in Figure 1B for a H1–Sal_20 kbp_ DNA stain. For comparison, the analysis of pure Sal_20 kbp_ DNA or H1 stains has been included in the Supporting Information (Figure S1–S2). We associate the PO_3_^−^ signal with the phosphate backbone of the DNA molecule, while the CNO^−^ signal is indicative of the total organic mass, i.e., histone and DNA. We used the Cl^−^ signal to trace salt stemming from the buffer. All three signals, CNO^−^, PO_3_^−^, and Cl^−^ were homogeneously distributed throughout the entire stain pattern indicating co‐deposition of the protein and DNA.

**Figure 2:**
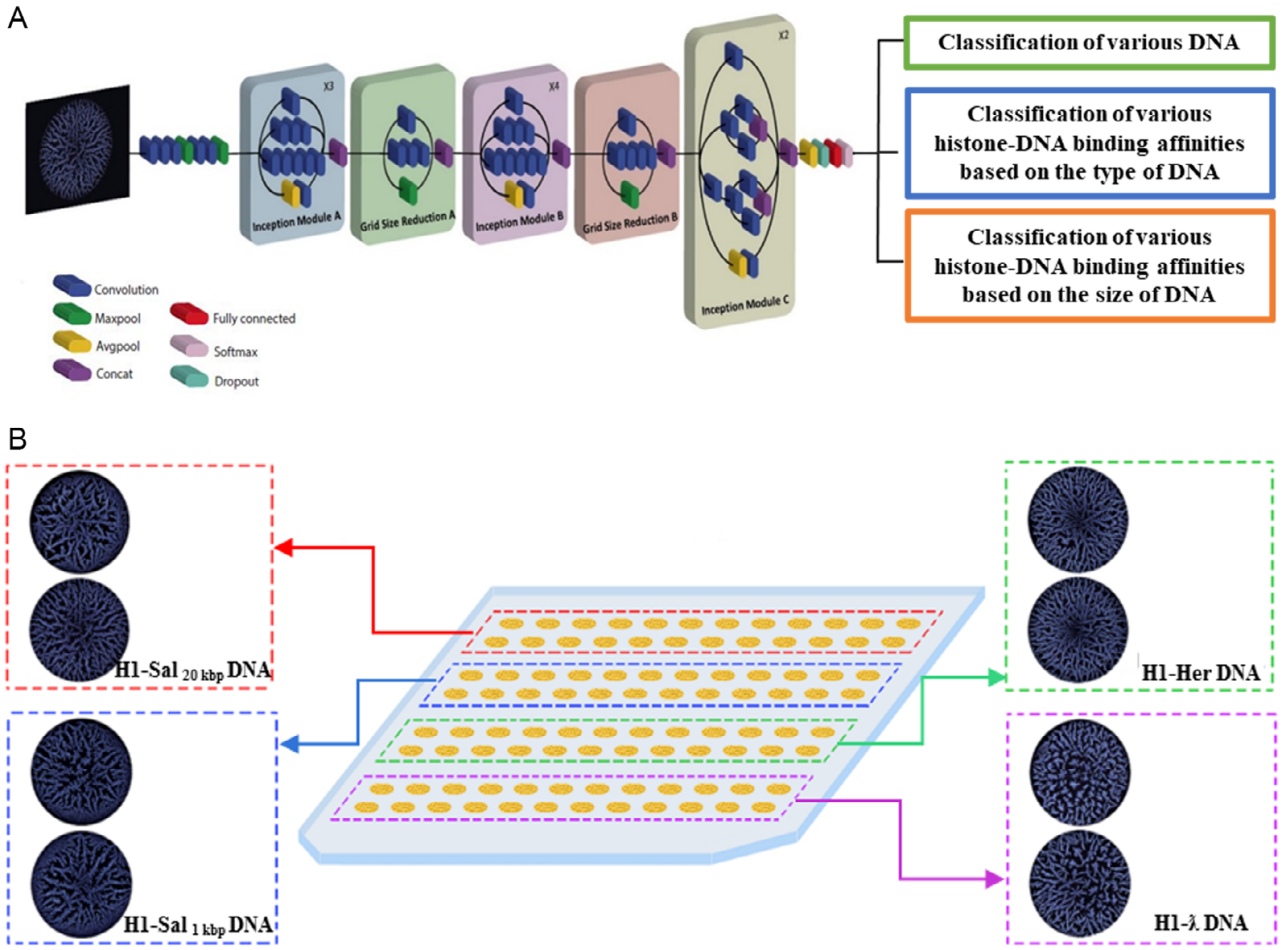
Classification of DNA, and H1‐DNA binding affinity based on the type and the size of DNA by analyzing their deposition patterns applying a DL approach. A) The pre‐trained deep convolutional neural network (CNN) was trained using a moderate number of PLM images depicting various groups of DNA and H1‐DNA complex stains, employing an established transfer learning technique. In the subsequent step, the CNN was tested with unseen images to evaluate its capability to classify different DNA and H1‐DNA mixtures based on the distinct patterns formed during their interactions. B) Representative PLM images of H1‐DNA stains from four different DNA deposited on a CVD‐coated glass slide, demonstrating pattern variability. Top to bottom: H1–Sal_20 kbp_ DNA (top, left), H–Her DNA (top, right), H1–Sal_1 kbp_ DNA (bottom, left), and H1‐ƛ DNA (bottom, right).

Next, we tested if the stain patterns can be utilized to classify diverse types of DNA of variant types and sizes (**Figure** **2**A). First, we generated arrays of drying droplet patterns obtained from four distinct DNA–histone (H1) mixtures (Figure 2B). For each group, we typically generate 400–500 PLM images, which are then used to train InceptionV3. InceptionV3 is a commercially available deep learning (DL) neural network.^[^ ^44^, ^49^ ^]^ We note that about 100 images can easily be recorded in 60 min.

### 2.1 Classification of Various DNA

**Figure 3:**
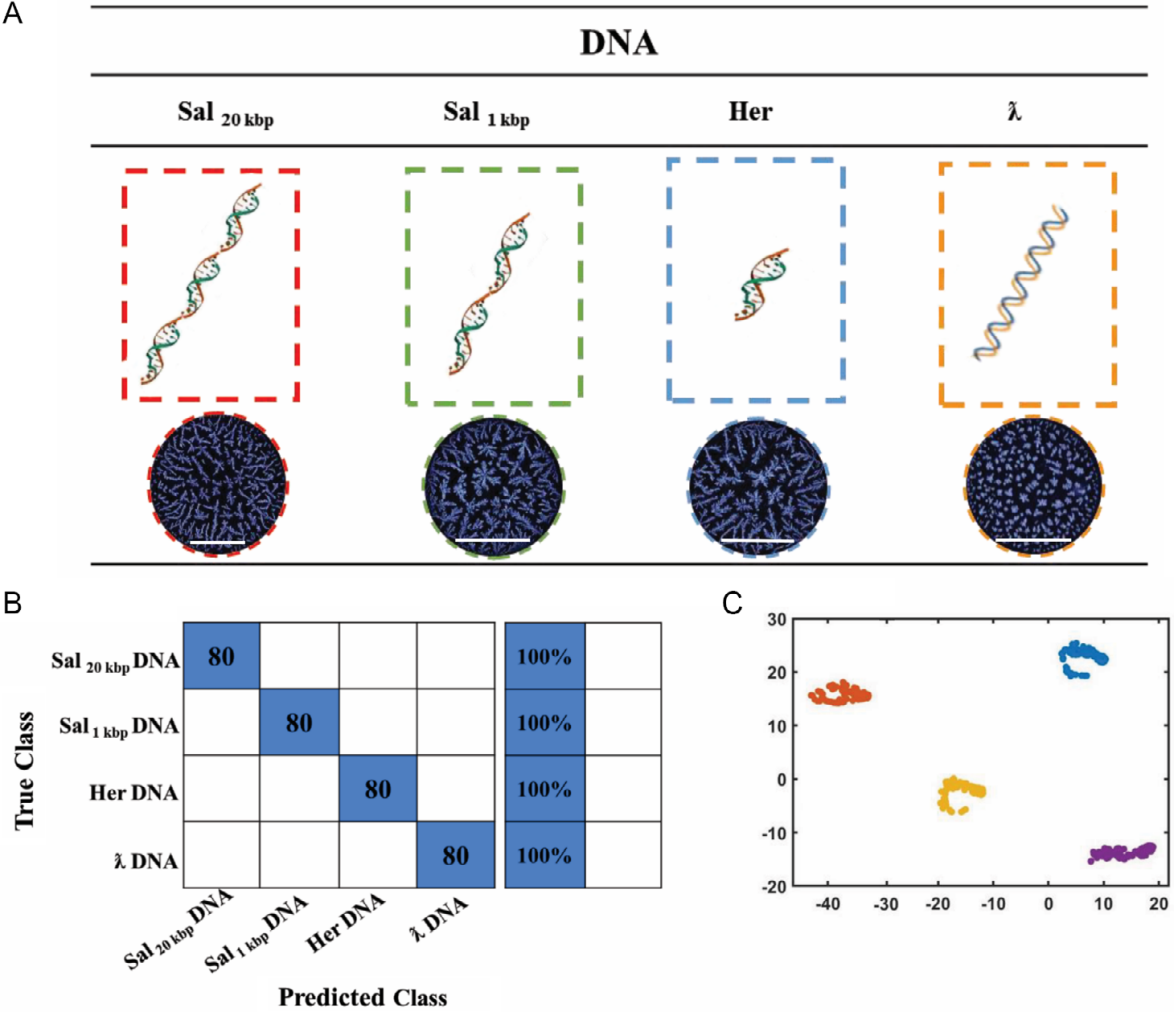
DL‐informed classification of various DNA. A) PLM images of Sal_20 kbp_ DNA (with red dashed‐line), Sal_1 kbp_ DNA (with green dashed‐line), Her DNA (with blue dashed‐line), and ƛ DNA (orange dashed‐line). The scale bars represent 1 mm. B) Four different DNA stains (varied by type and size) were analyzed using InceptionV3. C) t‐SNE plot displaying the results from the “SoftMax Activation” layer of the trained CNN. Sal_20 kbp_ DNA, Sal_1 kbp_ DNA, Her DNA, and ƛ DNA were indicated by purple, yellow, blue, and orange, respectively.

**Figure** **3**A displays the classification of PLM images of stain patterns containing Salmon DNA (Sal_20 kbp_), Sheared Salmon DNA (Sal_1 kbp_), Herring DNA (Her), and Lambda DNA (ƛ). These four types of DNA differ in their genetic information and/or molecular weight. Sal_20 kbp_ DNA, Sal_1 kbp_ DNA, and Her DNA constitute eukaryotic DNA, while ƛ DNA is of prokaryotic origin. Using the InceptionV3 DL network, the categorization of PLM images was carried out and resulted in an accuracy rate of 100% for each group, as illustrated by the confusion matrix presented in Figure 3B. In this case, the evaluation relied on training and validation dataset consisting of about 1600 images (400 images per group). For analysis, a test set comprising 320 (80 images per group) randomly selected images was used. All test set images were new and not previously included in the training set. Gradient‐weighted class activation mapping (Grad‐CAM) was utilized to generate activation maps of the softmax activation layer of the DL network. This approach is useful for identifying learning features associated with information‐rich regions of the PLM images.^[^ ^50^ ^]^ Figure S3 (Supporting Information) shows the heat map layers of the PLM images, underscoring the DL network's specific focus on central regions of the stain patterns. The application of the t‐distributed stochastic neighbor embedding (t‐SNE) algorithm (Figure 3C) after nonlinear dimensionality reduction in InceptionV3's “depth concatenation” layer revealed pronounced clustering for all four DNA variants. The t‐SNE visualizations were produced utilizing the “Softmax” layer of the CNN. The result of this layer is a four‐dimensional array composed of the spatial dimensions (*x* and *y*) of the images, the image channels, and the batch dimension, respectively.^[^ ^51^ ^]^ This observation further confirms that the stain patterns are not only distinguishable, but also predictive for a particular DNA type.

### 2.2 Relative Affinity of H1–DNA Interaction ‐ Based on DNA Type (Eukaryotic Versus Prokaryotic DNA)

A simple method for stratification of different protein–DNA complexes based on their binding affinity will be of significant interest to a wide range of applications. We utilized H1 as the DNA binding partner which was kept constant. We attempted to delineate the interactions between H1‐prokaryotic DNA (non‐specific binding) and H1‐eukaryotic DNA (specific binding). Following the above‐outlined experimental approach, the InceptionV3 model was then employed to classify different sets of protein–DNA mixtures, which varied in terms of the type of DNA and had different binding affinities.^[^ ^52^, ^53^ ^]^

**Figure 4:**
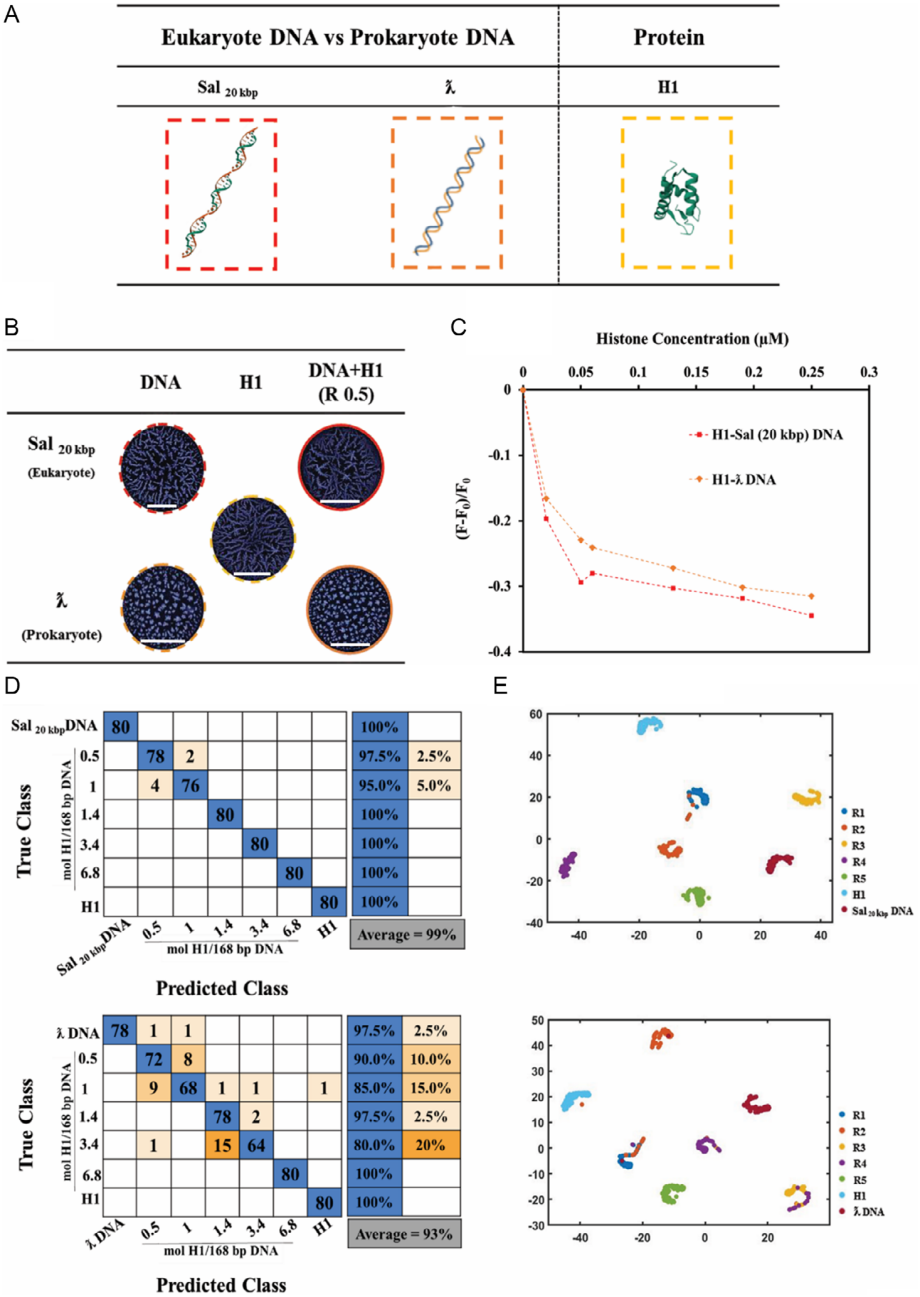
DL‐informed classification of eukaryote and prokaryote H1‐DNA interactions. A) Schematic representation of Sal_20 kbp_ DNA, ƛ DNA, and histone (H1). B) PLM images of Sal_20 kbp_ DNA (without histone), ƛ DNA (without histone), histone (without any DNA), and each H1‐DNA mixtures deposition patterns. The scale bars represent 1 mm. C) Ethidium bromide‐DNA complex displacement assay (each data point represents the average of samples obtained from two distinct experiments). The decrease in relative fluorescence intensity of the EtBr‐Sal_20 kbp_ DNA (red dash‐line), and EtBR‐ƛ DNA (orange dash‐line) complexes is a result of the interaction between H1 and each DNA. D) H1‐DNA mixture stains were analyzed using InceptionV3 for two different DNA which are mixed with histone. E) Visualization of the 'depth concatenation’ layer in the trained CNN.

Five distinct H1/DNA ratios (0.5–6.8 mole H1/168 base pairs DNA) were prepared for eukaryotic Sal_20 kbp_ DNA and the prokaryotic *ƛ* DNA. To exclude size effects, DNA with similar number of base pairs was procured from commercially available DNA sources (**Figure** **4**A). About 480 PLM images were collected for each ratio (400 images for training and validation sets and 80 images for the test set). The corresponding PLM images (Figure 4B) display distinct patterns for both, eukaryotic and prokaryotic DNA. The deposited patterns of eukaryotic DNA before and after adding the H1 appear similar. However, the patterns for H1‐prokaryotic DNA mixtures display visual characteristics that are distinct from those observed in the patterns of histone alone or prokaryotic DNA alone. Figure 4D depicts the classification of similar ratios of H1 with two different types of DNA (eukaryotic and prokaryotic) by the Inception V3 network. In Sal_20 kbp_ DNA samples exhibiting a higher binding affinity with H1, even minor quantities of histone result in affinity complexes as recognized by the CNN. We note that the associated changes in the stain patterns are not visible to the naked eye. The larger the structural changes, the clearer the CNN distinguishes the nature of the deposition patterns between different ratios of H1/DNA as indicated by higher prediction accuracy through CNNs.^[^ ^44^ ^]^ Figure 4D shows that the average prediction accuracy for various ratios of H1‐Sal_20 kbp_ DNA (eukaryotic) was 99%, exceeding the average prediction accuracy of 93% for H1‐ƛ DNA (prokaryotic).

According to the CNN‐derived confusion matrix, the prediction accuracies decreased with decreasing histone‐to‐base pair ratio and were the lowest for R0.5 and R1 (0.5 and 1 mol of H1 per 168 base pairs of Sal_20 kbp_ DNA). A total of 2.5% of stain images of the R0.5 group were misclassified as R1 group, while 5% of the R1 group were miscategorized as R0.5. The addition of more histone to Sal_20 kbp_ DNA (e.g., R1.4, R3.4, and R6.8) yielded accuracies of up to 100%. Conversely, the H1‐ƛ DNA (prokaryotic) mixtures demonstrated a lower average prediction accuracy of only 93%. The prediction accuracies for the groups of R0.5, R1, R1.4, and R3.4 were 90%, 85%, 97.5%, and 80%, respectively. Complete prediction accuracy (100%) occurred only at a ratio R6.8 H1‐ƛ DNA, while in the H1‐Sal_20 kbp_ DNA groups, this 100% prediction accuracy was achieved already at lower H1 concentrations at the ratio R1.4. The t‐SNE visualization (Figure 4E) showed clear clustering between different groups in H1‐ Sal_20 kbp_ DNA, bit not between different groups of H1‐ƛ DNA.

To corroborate the findings obtained from the CNN analysis, we conducted an ethidium bromide displacement experiment. Ethidium bromide typically binds to DNA through molecular intercalation. However, when DNA binds to a strong binding partner, the ethidium bromide molecule dislodges from the DNA, resulting in reduced fluorescence, due to rapid proton transfer from the excited singlet to water.^[^ ^54^ ^]^ The results of this assay are depicted in Figure 4C and clearly demonstrate that the incorporation of H1 into a pre‐incubated solution of Sal_20 kbp_ DNA‐EtBr resulted in a greater displacement of ethidium bromide compared to the solution with ƛ DNA‐EtBr.

### 2.3 Relative Affinity of H1–DNA Interaction ‐ The Role of DNA Size

**Figure 5:**
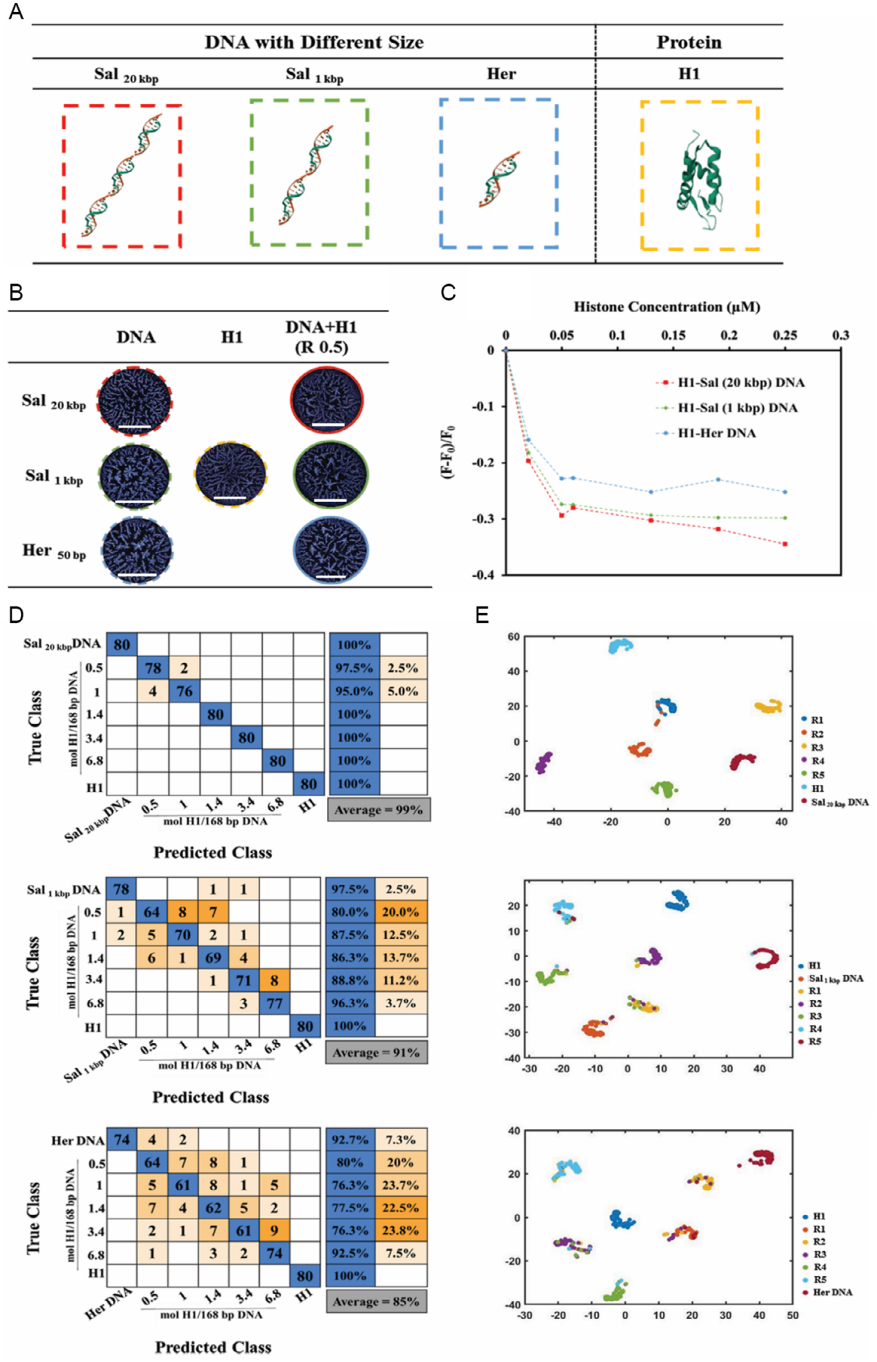
DL‐informed prediction of H1‐DNA interactions. A) Schematic representation of Sal _20 kbp_ DNA, Sal _1 kbp_ DNA, Her DNA, and H1. B) PLM images of each DNA (without histone), histone (without DNA), and each H1‐DNA mixture deposition pattern. The scale bars represent 1 mm. C) Ethidium bromide‐DNA complex displacement assay. D) H1‐DNA mixtures stains analyzed using InceptionV3. E) Visualization of the ‘depth concatenation’ layer in the trained CNN indicates different clusters corresponding to H1‐Sal _20 kbp_ DNA, H1‐Sal_1 kbp_ DNA, and H1‐Her DNA.

Longer DNA molecules provide more binding sites for H1, increasing the likelihood of H1–DNA interactions, and contributing to tighter compaction of DNA. The study of Renz^[^ ^21^ ^]^ found that the binding affinity to H1 varies for DNA with different fragment lengths, i.e., H1 showed a higher affinity for longer eukaryotic DNA fragments compared to shorter fragments. Moreover, Aviles et al.^[^ ^55^ ^]^ demonstrated that H1 exhibits a preferential binding affinity for high molecular weight calf thymus DNA compared to sheared DNA fragments. Changes in the molecular weight of DNA can influence the propensity of binding between DNA and protein, potentially altering the physical and chemical properties of the protein–DNA complex.^[^ ^56^ ^]^ In **Figure** **5**, Inception V3 was employed for classification of H1–DNA binding affinity based on the difference in the size of the DNA fragments. Three different sizes of eukaryotic DNA, including Sal_20 kbp_ DNA, Sal_1 kbp_ DNA, and Herring (Her) DNA (50 bp) were included in the comparison. Figure 5A shows a schematic representation of three distinct DNA sizes, while Figure 5B displays characteristic PLM images of the droplet stains that resulted from each complex. Using a training set of 360 PLM images, and a test set of 80 PLM images for each group (Figure 5D), we found that the average prediction accuracy from various ratios of H1‐Sal_20 kbp_ DNA was higher than the average prediction accuracy of H1‐Sal_1 kbp_ DNA and H1‐Her DNA complexes at similar histone ratios. With a completely unknown test set, i.e., none of the images has been previously encountered by the CNN, we observed 1%, 9% and 15% of total misclassification for H1‐Sal_20 kbp_ DNA, H1‐Sal_1 kbp_ DNA, and H1‐Her DNA, respectively. The H1‐Sal_20 kbp_ DNA ratios of 1.4 and higher resulted in highly distinct stain patterns, whereas in the case of the shorter DNA fragments, lower accuracies were observed. Furthermore, the t‐SNE plot (Figure 5E) showed various clusters corresponding to H1‐Sal_20 kbp_ DNA (with high binding affinity), H1‐Sal_1 kbp_ DNA (with medium binding affinity), and H1‐Her DNA (with low binding affinity). The trend in binding affinity was verified using the ethidium bromide displacement experiment described above (Figure 5C).

### 2.4 Classification of Unknown Histone–DNA Interactions

**Figure 6:**
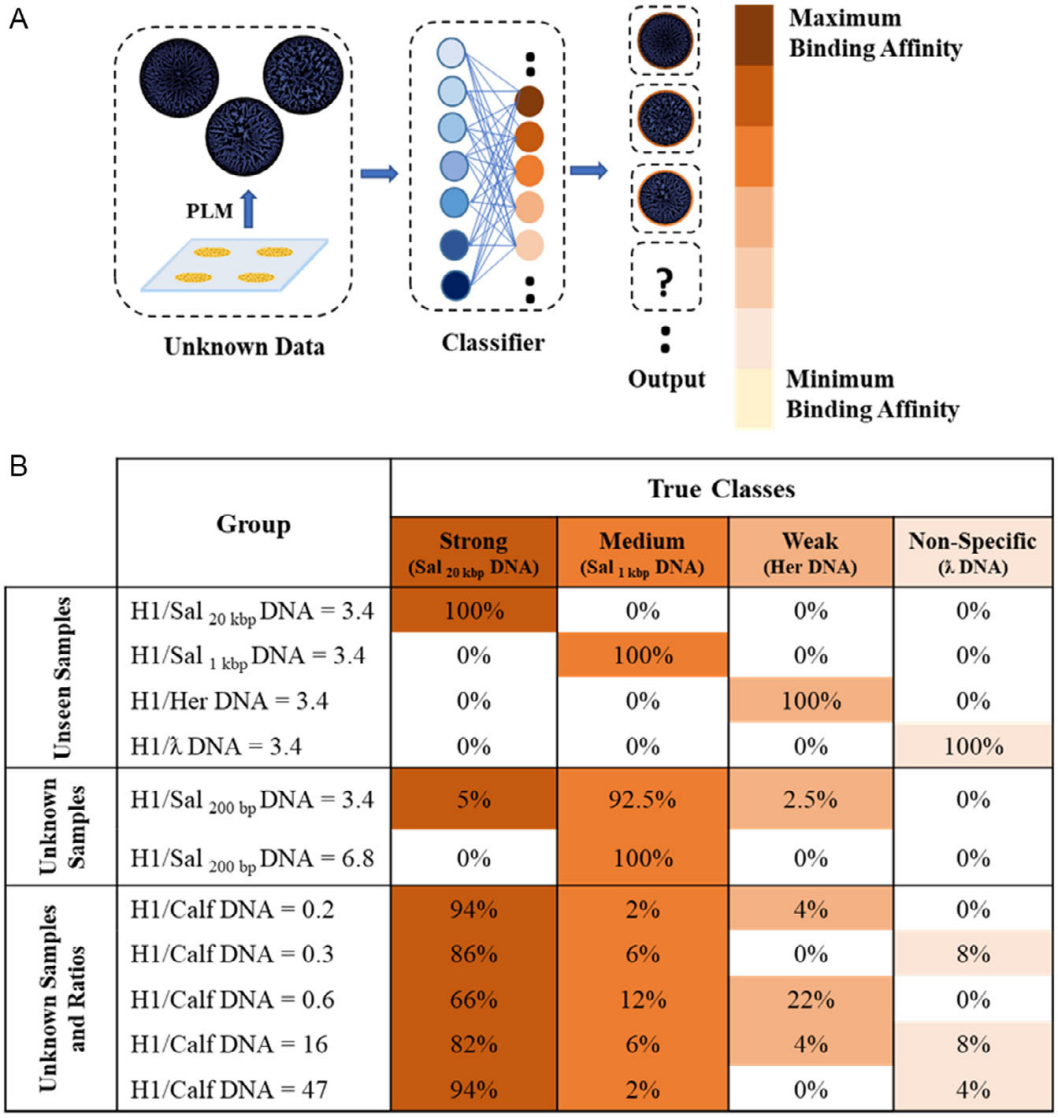
Examination of the pre‐trained network performance of DNA samples from a novel species (Calf DNA). A) Workflow for the classification of unknown samples. B) Prediction table for unseen/unknown protein/DNA samples.

Next, we wanted to assess the model's ability to predict different H1–DNA binding affinities of unknown DNA samples from species not included in the original training set. For this purpose, the neural network was trained with 25 distinct samples including various ratios of H1‐DNA for Sal_20 kbp_ DNA, Sal_1 kbp_ DNA, Her DNA, and ƛ DNA, DNA samples without H1, and H1 samples without DNA. The size of the training and validation set amounted to approximately ten thousand PLM images. This pre‐trained CNN was then used to predict various categories of H1‐DNA binding affinities from unknown DNA samples. **Figure** **6**A depicts the workflow used for the analysis of unseen/unknown samples, which involved data collection, feature extraction, and model training and validation. As shown in Figure 6B, the pre‐trained network accurately predicted the binding affinity of DNA from species included in the training set. We observed 100% prediction accuracy even though these images were unknown to the CNN (i.e., not included in the training set). For images of Calf DNA mixed with H1, samples were predominantly classified as strong binders (84.4%) with another 5.6% characterized as medium binders. Despite the fact that the CNN had never seen stain patterns that involve Calf DNA, the vast majority (96%) of stain patterns were accurately classified as eukaryotic DNA (i.e., specific H1 binding), while only 4% were misclassified as prokaryotic DNA (i.e., non‐specific binding). This high classification accuracy of DNA stain images from unknown DNA samples speaks to the robustness of our approach and demonstrates the model's capacity to evaluate unknown field data.

We evaluated how the prediction accuracy is influenced by the sample size of the test sets (Figure S4, Supporting Information). Based on only 10 images, the CNN classified 90% of the Calf DNA samples as strong binding. Further inclusion of additional images (e.g., 50, 80, and 100 images) increased the average prediction accuracy only marginally to 91%. These findings imply that based on only 10 images, reliable results can be achieved, underscoring the efficiency and feasibility of this ML approach.

## 3 Conclusions

Our study leverages deep‐learning techniques to investigate the relative binding affinity of DNA and H1. Specifically, analysis of stain images of histone–DNA complexes with the InceptionV3 CNN resulted in reliable and predictive categorization of their binding affinity. The network predicted that eukaryotic DNA interacts more strongly with H1 than prokaryotic DNA. With increasing fragment size, higher prediction accuracies were observed and trends between DNA fragment size and binding affinity to a histone were accurately predicted based on their stain patterns. When challenged with DNA from an unknown species (here Calf DNA), the CNN accurately classified the DNA as strong H1 binder. Interestingly, the cohort size of the test set had only marginal effects with a sample size suggesting that only 10 images are sufficient to obtain 90+% accuracy. While small sample sizes can be used for the test case, the robustness and reliability of the model still depend on a very large training data set. In this study, we generated ≈12 000 images in total that then were used to pre‐train the network to recognize unknown images. We also note that our study used four different DNA types and sizes, demonstrating that the model can generalize effectively beyond the initial simple cases.

The methodology developed in this study may facilitate the rapid screening of DNA‐binding candidates or protein–DNA interactions with potentially broad applicability in biotechnology and molecular biology. Further image screening with a diverse set of proteins and DNA is necessary to enhance the training of the network, leading to improved binding affinity predictions and generalization.

## 4 Experimental Section

4.1

4.1.1

##### Surface Preparation by CVD Polymerization

Glass slides with the dimensions of 120 mm × 80 mm and the thickness of 0.1 ± 0.05 mm (Optrovision, München, Germany) were cleaned by a plasma cleaner (Tergeo, Union city, CA, USA). Subsequently, they were coated with poly(*p*–xylylene) through a chemical deposition polymerization process.^[^ ^57^, ^58^ ^]^ In this process, the precursor [2,2]‐paracyclophane (Curtiss‐Wright Surface Technologies, Galway, Ireland), undergoes sublimation under reduced pressure and elevated temperature. Trough subsequent pyrolysis it is then transformed into *p*‐quinodimethane, which spontaneously polymerizes upon condensation onto a cooled surface.^[^ ^59^ ^]^ Argon was applied as a carrier gas at a flow rate of 20 sccm. The sublimation process occurred within a temperature range of 100–150 °C, succeeded by pyrolysis at 660 °C. The pressure for the coating procedure was maintained at 0.15 mbar.

##### Histone–DNA Solutions

Deoxyribonucleic acid sodium salt from salmon testes (Sal_20 kbp_), deoxyribonucleic acid from herring sperm (Her DNA), low molecular weight deoxyribonucleic acid from salmon sperm, and Histone H1 protein were purchased from Merck (Germany). Sheared Salmon (Sal_1 kbp_) DNA was produced through the sonication of Sal_20 kbp_ DNA, resulting in fragments with an average size of 1000 base pairs (Figure S5, Supporting Information). The sonication process took place on ice at a frequency of 20 kHz with an exposure time of 5 min. ƛ DNA was procured from Thermo Scientific. H1‐DNA complexes were prepared by the direct mixing of their previously equilibrated solutions in a binding buffer. This buffer contained 100 mM HEPES (pH 7.8) (Merck Chemicals GmbH), along with 150 mM potassium chloride (Merck Chemicals GmbH) and 50 mM ammonium sulfate (Merck Chemicals GmbH). Histone (H1) was gradually added to the DNA at five ratios: 0.5 (R0.5), 1 (R1), 1.4 (R1.4), 3.4 (R3.4), and 6.8 (R6.8) mole of histone (H1) per 168 base pairs of the DNA. The total mass concentration of H1 + DNA remained constant across all ratios. The solutions were gently stirred by an SB3 tube rotator (Stuart, Stone, UK) at 10 rpm for 60 min at room temperature (25 °C), and then stored at −20 °C until further use.

##### Droplet Deposition

An automated 96‐well microplate pipetting robot (epMotion 5070, Eppendorf AG, Hamburg, Germany) was used to load small droplet (2 μL) of the solutions on the coated surface by a 1‐channel‐dispenser (TS10, Eppendorf AG, Hamburg, Germany). The pipetting system was set up to put 96 droplets on each glass plate, forming a grid with 12 columns and 8 rows. To control the evaporation rate, the robot was inserted inside a climate chamber (ICH 750, Memmert GmbH + Co. KG, Schwabach, Germany) with the controlled temperature of 23 ± 0.5 °C and a humidity of 40 ± 3%. After drying the droplets (≈50 min). Deposition pattern images were acquired using an Olympus polarizing optical microscope with the exposure time of 1/20 s (BX‐53F, Tokyo, Japan) equipped with an automated stage. A 4 K digital camera (UC90, Münster, Germany) operating in color profile mode captures images by assigning intensity values between 0 and 255 to each pixel for the red, green, and blue (RGB) channels. All the images were taken under the same microscope settings, using a 10× magnification lens, and stitched together using the Multi Image Alignment (MIA) algorithm from CellSens software (Olympus, Tokyo, Japan).^[^ ^44^ ^]^ Each dried droplet's image had a size of 2344 pixels × 1878 pixels in JPG format.

##### Convolutional Neural Network of Training and Testing Set Images

The CNN training and further processing of the PLM images were performed using MATLAB software (R2022a, MathWorks Inc.). The Inception V3 (the pre‐trained CNN network) was selected to train and test images, due to its high response speed and acceptable accuracy. The Inception V3 network is a deep convolutional neural network architecture primarily designed for image classification tasks. It is an evolution of the original Inception model, which was developed by Google. Inception V3 is characterized by its use of “Inception modules,” which are designed to efficiently capture multi‐scale features in an image. It contains 315 layers, making it a relatively deep neural network. The network requires input images with three color channels corresponding to RGB to be resized to 299 pixels × 299 pixels. This means that the images used for training and inference should be preprocessed to this size before being fed into the network. The images were cropped to 1878 × 1878 pixels by trimming 233 pixels from each side and then resized to fit the CNN's input layer requirements. The preprocessing step involves “rescale‐symmetric” normalization the pixel values to [−1 1] range. The output parameters are the probabilities associated with each class in the classification task. Using the same format for all groups ensured consistent comparisons and maintained the integrity of our analysis. The resizing of all images to 299 × 299 pixels ensured standardized input and minimized the impact of the initial image format on the final analysis. Using a transfer learning approach, a network initially pre‐trained on a substantial dataset of image features underwent fine‐tuning with a relatively small set of new images.^[^ ^60^, ^61^ ^]^ In the transfer learning process, the final classification layer was excluded from the network and then retrained with the new dataset. The fine‐tuning process involved adjusting the parameters across all layers with a consistent global learning rate of 0.001, a minimum batch size of 32 images, and a maximum of 40 epochs. During training, ≈360 images were used per class, while 40 images per class served as validation data. For evaluating the trained network, 80 images per class were used as a separate test set. Importantly, there was no overlap between the training, validation, and test sets to ensure unbiased evaluation.^[^ ^44^ ^]^ The t‐distributed Stochastic Neighbor Embedding (t‐SNE) algorithm, known for visualizing high‐dimensional data, was utilized on the last hidden layer representation of the trained CNN (softmax) to demonstrate the network's ability to cluster different groups effectively. This t‐SNE analysis was carried out using a MATLAB ML package, employing a perplexity of 30 and a learning rate of 500. To gain insights into the regions of the image that significantly influence the deep learning network's classification decisions, the visualization algorithm called gradient‐weighted class activation mapping (Grad‐CAM) was employed.

##### SEM

The structural characteristics of the H1–DNA complex and salt of stain droplets were analyzed through scanning electron microscopy (SEM) using a TESCAN VEGA3 instrument. To mitigate surface charging effects, a fine layer of gold was sputtered onto the samples before SEM imaging. The secondary ion detector was used. All SEM images were acquired at an electron accelerating voltage of 15 kV with a working distance of 7.2 mm.

##### TOF‐SIMS

Time‐of‐flight secondary‐ion mass spectrometry (TOF‐SIMS) was performed using a mass spectrometer (ION‐TOF GmbH, Münster, Germany) equipped with a Bi cluster liquid metal primary‐ion source and a non‐linear time‐of‐flight analyzer. For spectrometry, short primary‐ion pulses (<1 ns) of the Bi source was operated in the “bunched” mode providing Bi^1+^ ion pulses at 25 keV energy and a lateral resolution of 5 μm. As the droplets were larger than the maximum deflection range of the primary‐ion gun of 500 × 500 μm^2^, the images were obtained using the manipulator stage scan mode. Negative polarity spectra were calibrated on the C^−^, CH^−^, and CH^2−^ peaks. Spectrometry was performed in static SIMS mode by limiting the primary‐ion dose to <10^11^ ions cm^−2^. Charge compensation was necessary because of the glass substrate so that an electron flood gun providing electrons of 20 eV was applied and the secondary‐ion reflectron tuned accordingly.

##### Ethidium Bromide Displacement Assay

The ethidium bromide (EtBr) (VWR, USA) displacement assay was performed according to Geall et al. (2000) in 10 mM HEPES buffer (pH 7.8) along with 15 mM KCl and 5 mM (NH_4_)_2_ SO_4_.^[^ ^54^ ^]^ Steady state fluorescence measurements were done on a spark multimode reader from Tecan (TECAN, Deutschland GmbH).

The 96‐well black well plate was maintained at a temperature of 293.15 K. The working volume of the solution (200 μL) contained 0.1 m EtBr for each 168 base pairs of DNAs. A solution of EtBr was introduced into the stirring solution and allowed to equilibrate for 15 min. Subsequently, aliquots of histone were added to the stirring solution, and fluorescence was measured after 30 min of equilibration.

##### Statistical Analysis

The analysis of variance was performed to examine the impact of the quantity of unseen samples on prediction accuracy, utilizing the least significant difference (LSD) method with SAS 9.1.3 software (SAS Institute, Inc., 1999, Cary, NC, USA).The LSD method was applied to identify significant differences with a significance level set at *p* < 0.05 (Montgomery and Runger, 2011).^[^ ^62^ ^]^

## Conflict of Interest

The authors declare no conflict of interest.

## Supporting information

Supplementary Material

## Data Availability

The data that support the findings of this study are available from the corresponding author upon reasonable request.
