# Supplementary material for: Deep Learning‐Based Classification of Histone–DNA Interactions Using Drying Droplet Patterns
Source: Small Sci. 2024 Aug 10;4(11):2400252. doi: 10.1002/smsc.202400252 (PMC11935254; doi:10.1002/smsc.202400252)
Supplement: Supplementary file 1 — Supplementary Material [file SMSC-4-2400252-s001.zip › smsc.202400252-sup-0001-suppdata-S1_1.pdf]

Supporting Information

Deep learning-based classification of Protein-DNA interactions using drying droplets patterns

Safoura Vaez[1], Bahar Dadfar[1], Meike Koenig[1], Matthias Franzreb[1], Joerg Lahann[1,2*]

[1] Institute of Functional Interfaces (IFG), Karlsruhe Institute of Technology (KIT), Hermann-von-Helmholtz-Platz 1, 76344 Eggenstein-Leopoldshafen, Germany

[2] Biointerfaces Institute, Departments of Chemical Engineering, Materials Science and Engineering, and Biomedical Engineering, and the Macromolecular Science and Engineering Program, University of Michigan, Ann Arbor, MI 48109, USA

** Corresponding author: Prof. J. Lahann; Email: lahann@umich.edu*

Video S1) Drying process of a H1-Sal $_{20\ kbp}$ DNA droplet

The video illustrates a droplet containing a solution of H1-Sal $_{20\ kbp}$ DNA drying on a poly(p-xylylene) (PPX) coated surface under polarized light. Drying and recording were conducted under standard laboratory conditions for a droplet with a volume of 2 µL. The video is sped up twelve times its original speed.

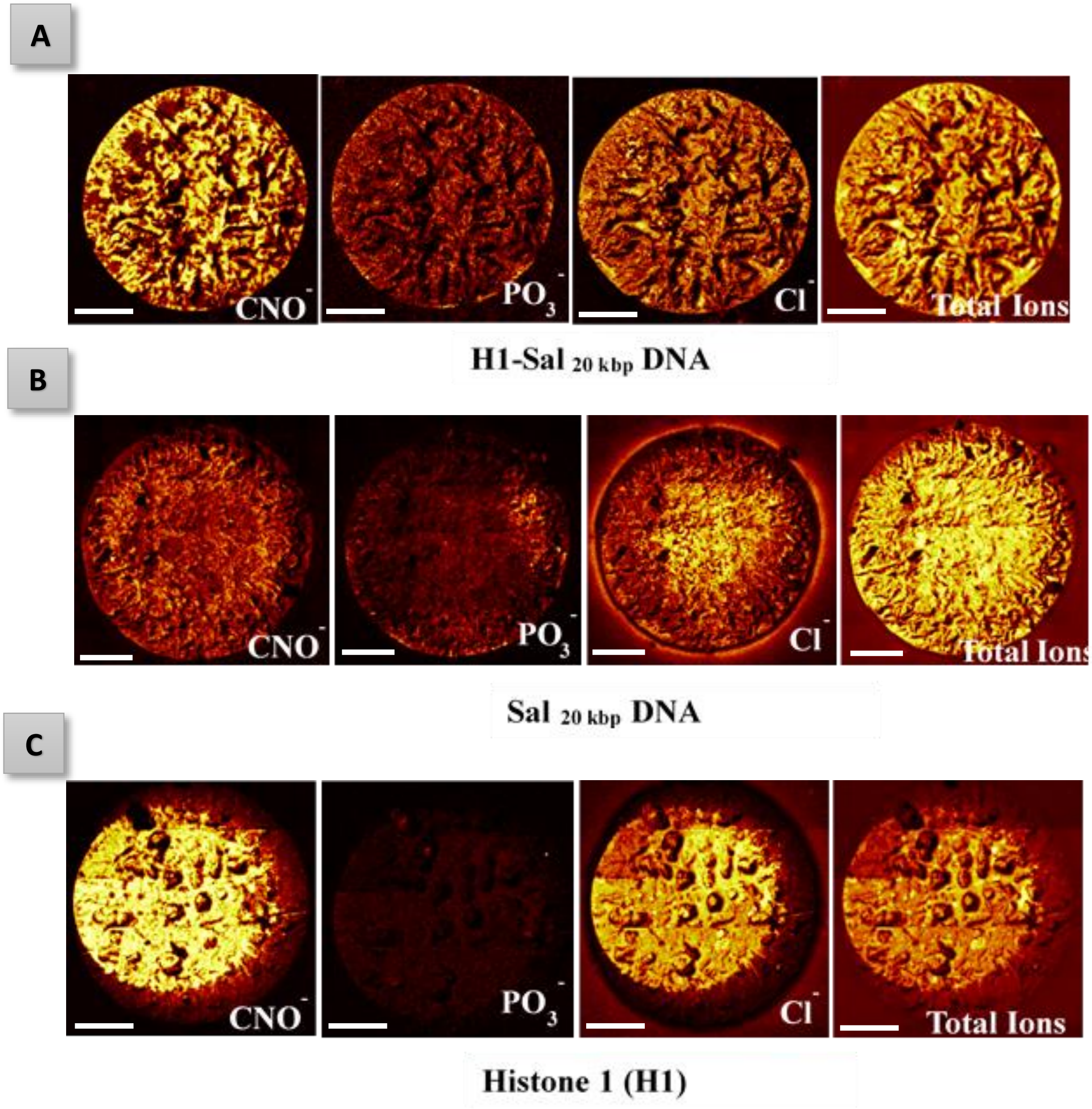

Figure S1) TOF-SIMS imaging.

Brown scale images of A) H1-Sal $_{20\ kbp}$ DNA sample: Amino acids of protein and base pairs of DNA were identified by CNO^- (scale 0-30), the backbone of DNA was identified by PO_3^- (scale 0-2), and buffer crystals were identified by Cl^- (scale 0-30); B) Sal $_{20\ kbp}$ DNA sample: CNO^- (scale 0-20), PO_3^- (scale 0-10), Cl^- (scale 0-30) and C) H1 (histone1) sample: CNO^- (scale 0-30), PO_3^- (scale 0-2), Cl^- (scale 0-30). All total ion images (scale 0-1300) represent the combined signals from all detected ions, displaying the variation in detected ionization over the droplet area. All scale bars represent 500 µm

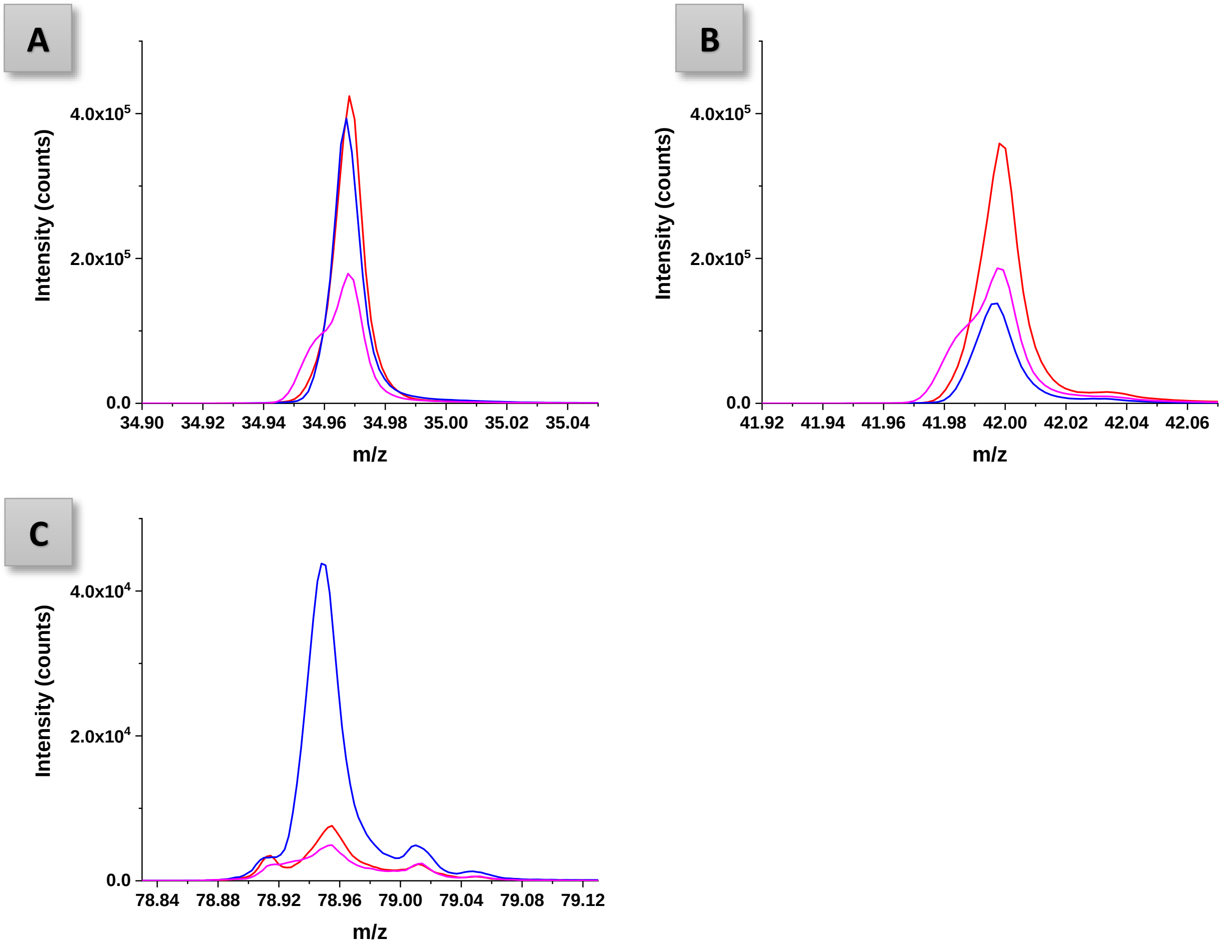

Figure S2) TOF-SIMS spectra.

Characteristic spectral areas, corresponding to the images in Figure S1, displaying the total areal intensity of A) Cl^-, B) CNO^- and C) $PO3^-$. The red, blue, and pink spectra represent histone (H1), Sal $_{20\ kbp}$ DNA, and H1+ Sal $_{20\ kbp}$ DNA mixture samples, respectively.

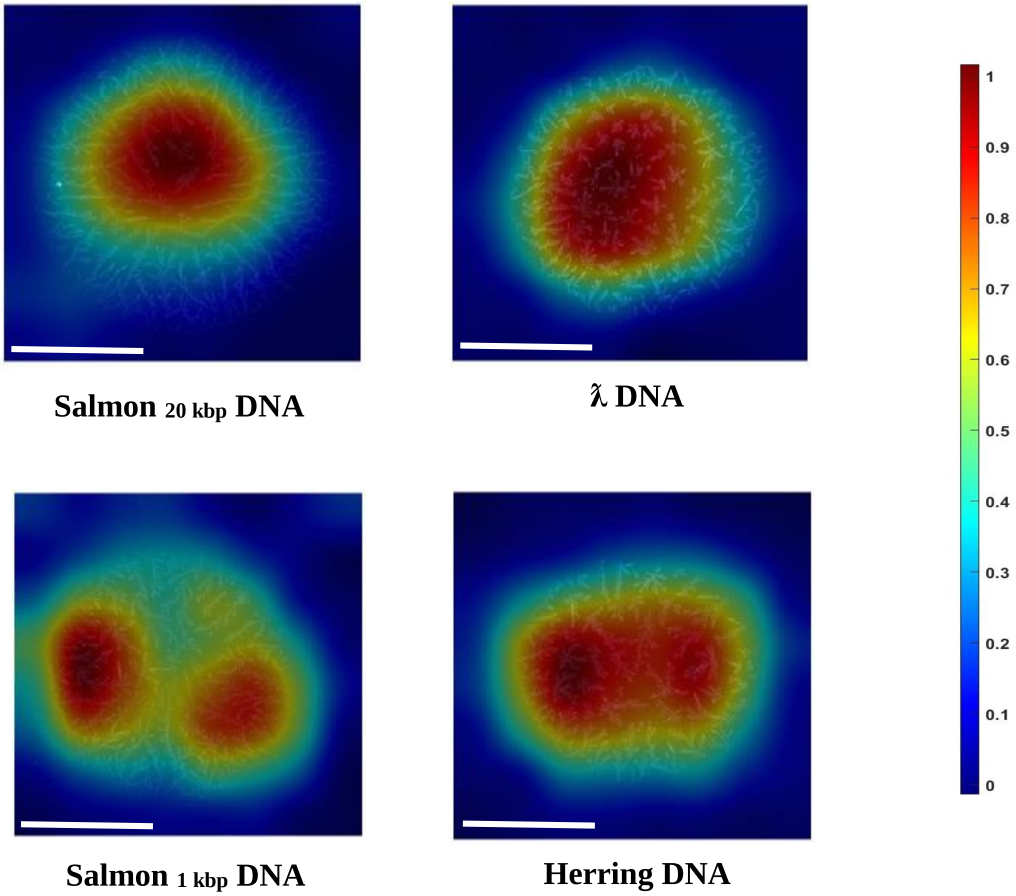

Figure S3) Grad-CAM activation maps

Activation maps of the final convolutional layer of the DL network were generated using Gradient-weighted class activation mapping (Grad-CAM) to identify features of discriminatory regions in the PLM images. Heat map layers of the PLM images depicting deposition patterns of different DNA were created. All images were accurately classified by the trained network, which utilized the InceptionV3 DL network. The scale bars represent 1 mm.

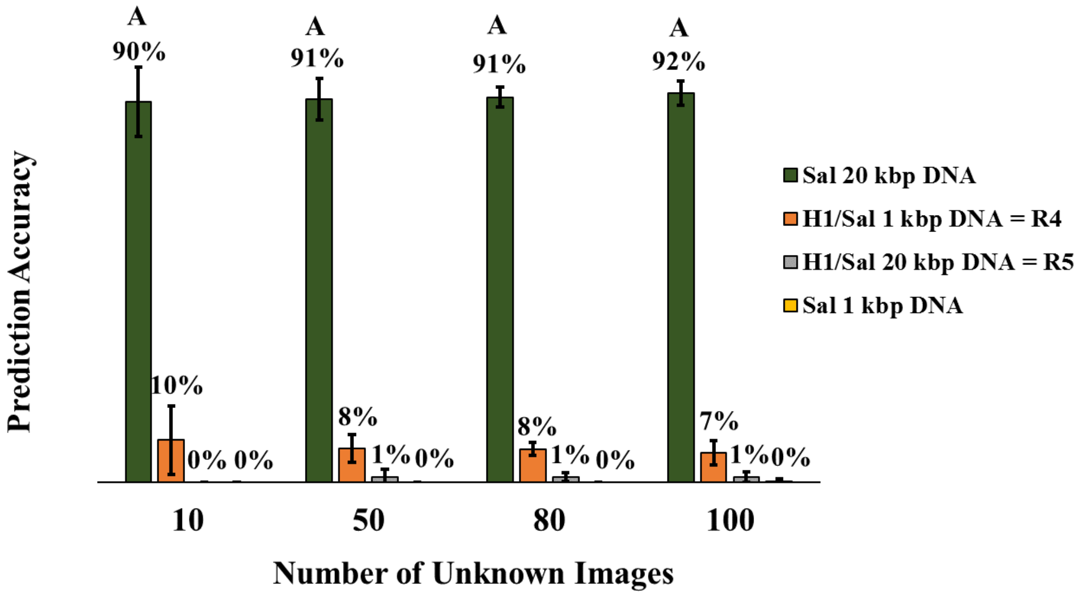

Figure S4) The impact of varying the quantity of unseen test images on the prediction accuracy of the trained neural network. The error bars in the graphical representation indicate the standard deviation (N=3). Groups sharing identical letters indicate no statistically significant differences based on least significant difference (LSD)-adjusted comparisons.

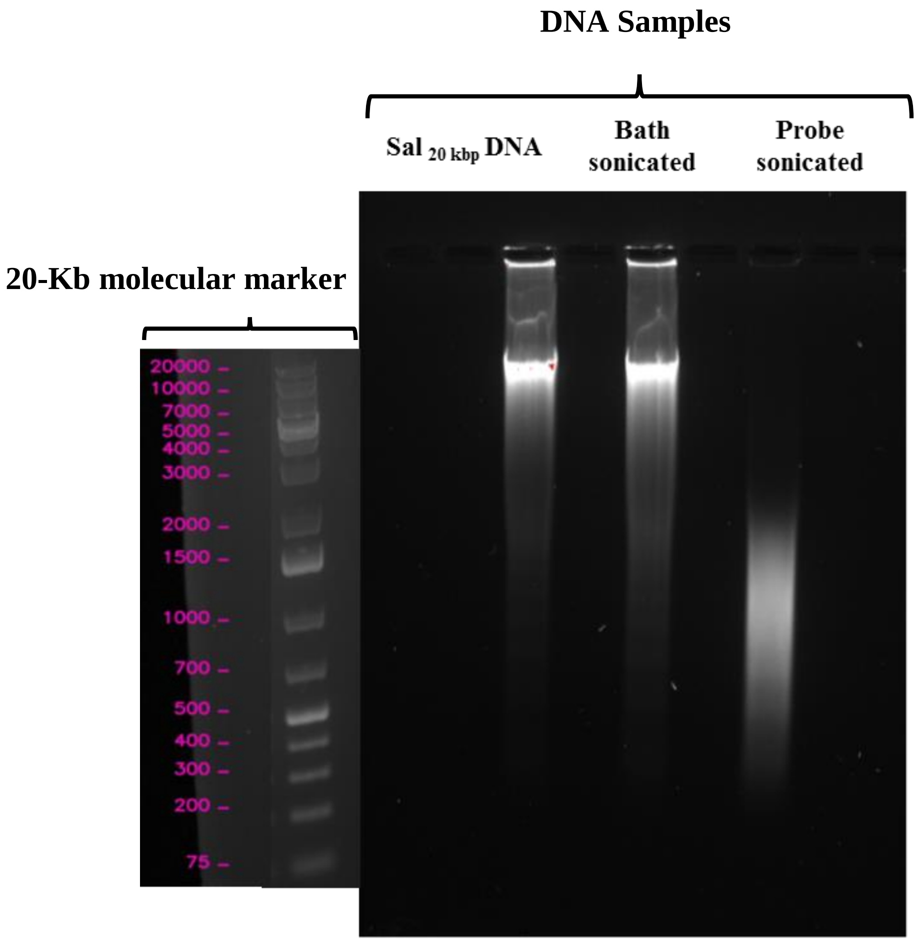

Figure S5) Agarose gel electrophoresis

Agarose gel electrophoresis (1%) for Sal $_{20\ kbp}$ DNA, sonicated Sal $_{20\ kbp}$ DNA (bath sonication for 5 second, and direct probe sonication for 5 min with 20 kHz. The determination of fragment sizes is based on their positioning on the gel compared to the molecular marker.
